# HLA-E-Directed Accumulation of KIR^+^NKG2C^+^ NK Cells upon HCMV Peptide Presentation In Vitro: Association with the Ex Vivo Phenotype

**DOI:** 10.3390/ijms27136087

**Published:** 2026-07-07

**Authors:** Nadezhda A. Alekseeva, Maria O. Ustiuzhanina, Maria A. Streltsova, Julia D. Vavilova, Alexander M. Sapozhnikov, Elena I. Kovalenko

**Affiliations:** 1 Shemyakin and Ovchinnikov Institute of Bioorganic Chemistry, Russian Academy of Sciences, 117997 Moscow, Russia; nadalex@inbox.ru (N.A.A.); mashaust1397@gmail.com (M.O.U.); mstreltsova@mail.ru (M.A.S.); juliateterina12@gmail.com (J.D.V.); amsap@mail.ru (A.M.S.); 2 Institute of Translational Medicine, Pirogov Russian National Research Medical University, 117887 Moscow, Russia; 3 Center for Molecular and Cellular Biology, 121205 Moscow, Russia; 4 Dmitry Rogachev National Research Center of Pediatric Hematology, Oncology and Immunology, 117997 Moscow, Russia

**Keywords:** NK cells, HCMV, NKG2A, NKG2C, inhibitory KIRs, HLA-DR, HLA-E, solid tumors, HCMV peptides

## Abstract

NK cells co-expressing NKG2C and inhibitory KIRs represent promising effector candidates when directed against HLA-E^+^ tumors. These cells may propagate in vitro upon HCMV peptide (LFL) presentation within HLA-E by interacting cells due to KIR^+^NKG2C^+^ cell expansion or via NKG2C or KIR de novo expression. This study aimed to clarify whether these accumulation mechanisms are connected to the healthy individual’s ex vivo NK cell phenotype. The architecture of the phenotype was characterized by the distribution of markers known to be associated with NK cell differentiation and the HCMV adaptive response, including NKG2C, KIR2DL2/3, and HLA-DR. We also analyzed NK cells that differed in marker expression cultured with or without LFL in vitro. The most pronounced NKG2C expression de novo was observed in KIR^+^ NK cells, associated with a higher HLA-DR^+^ ex vivo proportion. Enhanced LFL-dependent KIR^+^NKG2C^+^ cell expansion of the CD57^−^ fraction was associated with a high content of adaptive NK cells ex vivo, while the CD57^+^KIR^+^NKG2C^+^ expansion rate correlated with a high level of NKG2A ex vivo. Moreover, LFL-activated cultures more effectively eliminated HLA-E^+^ tumor spheroids. These data are of interest both for KIR^+^NKG2C^+^ NK cell therapeutic application and for widening understanding of virus-induced NK cell repertoire modulation.

## 1. Introduction

The population of NK cells, the innate immune lymphocytes, contains subsets of diverse phenotypic features and functions. A wide variety of receptors recognizing pathogenic and tumor-associated molecular patterns draws special attention to NK cells in the context of antitumor therapy. In particular, the adaptive NK cell subset is characterized by a long lifespan and pronounced cytotoxic potential [1,2]. Accumulation of such cells in peripheral blood is observed during viral infections. The best-studied to date are HCMV-specific adaptive NK cells with the CD57^+^NKG2C^+^NKG2A^−^ phenotype [3,4]. The ligand of the activating NKG2C receptor is the HLA-E molecule, which presents the HLA class I leader sequence or relevant peptides of viral origin [5,6,7].

HLA-E expression may serve as a marker of healthy cells that should be protected from the NK cells’ cytotoxicity, as well as a system for virally infected or cancer-transformed cells to evade immune surveillance [8,9,10]. HLA-E surface overexpression on target cells leads not only to NKG2C-mediated activation, but also to suppression of NK cell activity via the inhibitory receptor NKG2A [11,12]. It is noted that NKG2A-blocking antibody therapy can significantly improve the effectiveness of anticancer treatment [13].

However, NKG2A is not only a checkpoint but also a marker of NK cell licensing, cytotoxic maturity, and the ability to accumulate granzyme B and perforin in cytotoxic granules to eliminate targets [14].

NKG2A expression is characteristic of less differentiated NK cells, whereas more differentiated cells, including adaptive NK cells, express other licensing markers—inhibitory killer-cell immunoglobulin-like receptors (KIRs), whose ligands are the HLA-A, -B, and -C molecules, but not HLA-E [15]. KIR expression is frequently observed in the adaptive CD57^+^NKG2C^+^ cell subset [16,17]. It is also known that during HCMV infection in vivo, the expansion of NKG2C-positive cells is observed due to the growth of individual KIR^+^ clones [18].

NK cells co-expressing KIRs and NKG2C are a promising basis for generating effectors directed at HLA-E-expressing targets. It has already been shown that KIR^+^NKG2C^+^ cells obtained from superdonors are capable of effectively eliminating acute myeloid leukemia cells [19]. Nevertheless, the accumulation of such cells in donors without a significant subset of adaptive NK cells is understudied.

Yet, one potential obstacle to obtaining KIR^+^NKG2C^+^ effector cells on the scale required for therapy is the high degree of differentiation of these cells, the low levels of cytokine receptors, and, consequently, reduced proliferative activity in vitro. NKG2C^+^ cell expansion during HCMV infection is achieved through the HCMV peptide presentation within HLA-E on the infected cells’ surface [7,20]. One approach to the accumulation of such cells is NKG2C-mediated stimulation through the presentation of the HLA-E+peptide complex on the surface of feeder cells. It was previously shown that the presentation of the VMAPRTLFL (LFL) peptide within HLA-E leads to NKG2C^+^ cell accumulation in vitro [21,22], including KIR-expressing cells [23]. We also demonstrated that LFL presentation to CD57^−^ NK cells leads to their increased proliferative activity and identified a link between proliferation intensity in cultures and the proportion of HLA-DR^+^CD56^bright^ cells ex vivo [24]. In the context of HCMV infection, HLA-DR expression on the NK cell surface is not only an activation marker but also a tool for communicating with the adaptive immune system. As non-classical antigen presenters, NK cells may present HCMV peptides within HLA-DR molecules to T cells, activating and engaging them in the immune response [25]. However, the link between LFL-mediated expansion and the HLA-DR^+^CD56^bright^ subset remains unclear.

In this study, we investigated the NK cell response to HCMV (LFL) peptide presentation in vitro in the context of the accumulation of cells co-expressing KIRs and NKG2C, both in bulk cultures and in cultures of isolated cell subsets with different expression of KIR, NKG2C, and the maturity marker CD57. In the obtained cultures, we assessed the proliferative activity, phenotypic plasticity, cytokine-dependent IFNγ production, and natural cytotoxicity, focusing on the associations of these cultures’ properties with the NK cell ex vivo phenotype, characterized by NKG2C, KIR2DL2/3, HLA-DR, and NKG2A. The results indicate that both mechanisms—the proliferation of cells with the same phenotype and de novo expression of KIR and NKG2C—are realized for the peptide-associated accumulation of KIR^+^NKG2C^+^ cells during HCMV infection. We showed that the inclination of KIR^+^NKG2C^+^ cells to a particular pathway of accumulation, as well as the plasticity of their phenotype and features of their functional profile, are associated with the expression of HLA-DR, KIR, NKG2C, and NKG2A markers in an NK cell population ex vivo. An important aspect of this study was assessment of the ability of the LFL-stimulated NK cells to eliminate HLA-E-expressing tumor cells in 3D cultures. These results have direct implications for donor selection in KIR^+^NKG2C^+^ NK cell-based therapy of HLA-E-positive solid tumors.

## 2. Results

### 2.1. Diverse Architecture of NK Cell Phenotype Based on Ex Vivo HCMV-Associated Marker Expression

**Figure 1:**
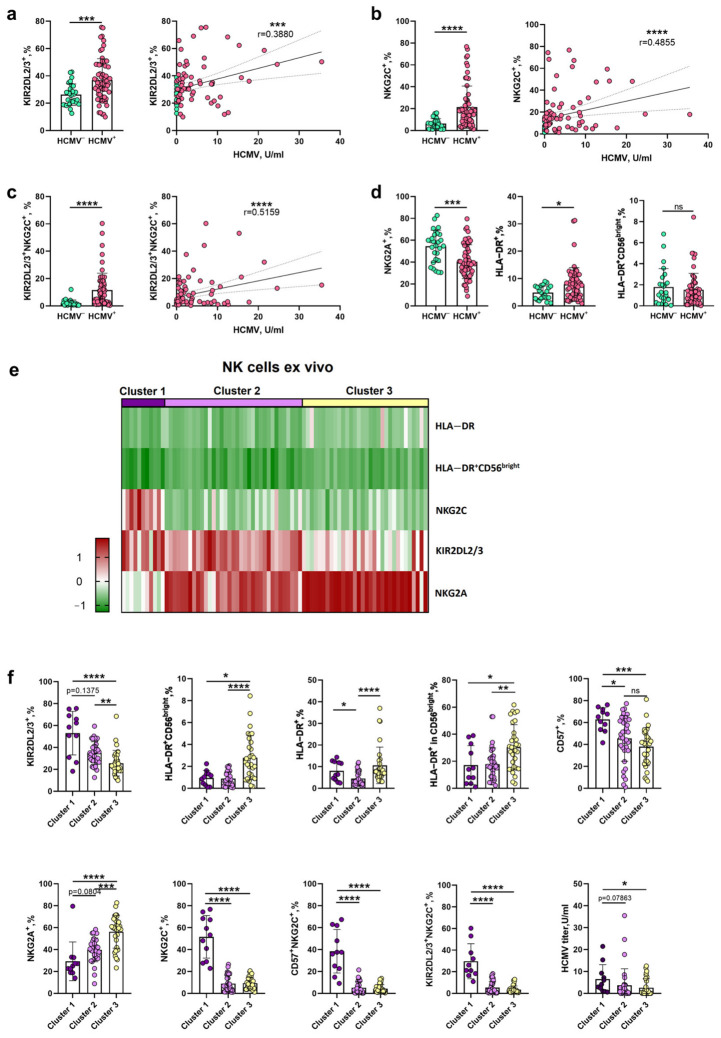
NK cell phenotype ex vivo: (**a**) the proportion of KIR^+^ cells in total NK cell population of HCMV^−^ and HCMV^+^ individuals (left), correlation between the proportion of KIR^+^ ex vivo and the titer of antibodies to HCMV (right); (**b**) the proportion of NKG2C^+^ cells in the NK cell population of HCMV^−^ and HCMV^+^ individuals (left), correlation between the proportion of NKG2C^+^ ex vivo and the titer of antibodies to HCMV (right); (**c**) the proportion of KIR^+^NKG2C^+^ cells in the NK cell population of HCMV^−^ and HCMV^+^ individuals (left), correlation between the proportion of KIR^+^NKG2C^+^ ex vivo and the titer of antibodies to HCMV (right); (**d**) the proportions of NKG2A^+^, HLA-DR^+^, and HLA-DR^+^CD56^bright^ cells in the NK cell population of HCMV^−^ and HCMV^+^ individuals; (**e**) hierarchical tree clustering of HCMV^+^ donors based on HLA-DR^+^, HLA-DR^+^CD56^bright^, NKG2C^+^, NKG2A^+^, KIR2DL2/3^+^, CD57^+^NKG2C^+^, and KIR2DL2/3^+^NKG2C^+^ proportions in NK cell population, Ward’s method, standardized data; (**f**) the proportions of HLA-DR^+^CD56^bright^, KIR2DL2/3^+^, HLA-DR^+^, CD56^bright^, NKG2A^+^, NKG2C^+^, CD57^+^NKG2C^+^, and KIR2DL2/3^+^NKG2C^+^ cells in the NK cell population of different clusters’ donors. Number of donors: N = 91 (HCMV^+^-67, HCMV^−^-24). Statistical analysis was performed using nonparametric Mann–Whitney U test (**a**–**d**) or nonparametric Kruskal–Wallis test followed by Dunn’s multiple comparison post hoc test (**f**) (* *p* < 0.05, ** *p* < 0.01, *** *p* < 0.005, **** *p* < 0.001, ns—not significant); means ± SD are shown. Correlation analysis was done using Pearson’s correlation; *p* < 0.05 was considered statistically significant.

We first investigated the relationship between the serological status of individuals with respect to HCMV infection and the expression of inhibitory receptors KIR2DL2/3 on the NK cell surface. For phenotyping, we selected 91 individuals (67 HCMV-seropositive and 24 HCMV-seronegative) carrying one (C2/C1) or two (C1/C1) HLA-C1 alleles, as well as the KIR2DL2 and/or KIR2DL3 genes. KIR2DL2/3-expressing cells (hereafter referred to as KIR^+^) were thus considered to have undergone licensing and to possess high cytotoxic potential [14]. We demonstrated that the NK cells of HCMV-seropositive individuals contained a larger fraction of KIR^+^, NKG2C^+^, and KIR^+^NKG2C^+^ cells, and that the proportion of these cells showed a low positive correlation with the HCMV IgG antibody titer (Figure 1a–c). The proportion of KIR^+^ cells was independent of the number of HLA-C1 alleles, whereas a trend toward a higher proportion of KIR^+^NKG2C^+^ cells in the NK cell pool was observed in donors carrying two HLA-C1 alleles (Figure S1a,b).

In addition to NKG2C, HCMV infection is known to affect the expression of several other markers. In the NK cell compartment of HCMV-seropositive individuals in this cohort, a reduced proportion of NKG2A^+^ cells and an increased proportion of HLA-DR^+^ cells were observed (Figure 1d). We have previously shown that a higher proportion of HLA-DR^+^CD56^bright^ cells within the total NK cell population is associated with more intensive proliferation of CD57-negative NK cells in response to HCMV peptide (LFL) presentation [24]. However, no difference in the proportion of HLA-DR^+^CD56^bright^ cells within NK cells was detected between HCMV-seropositive and HCMV-seronegative individuals (Figure 1d). Since the HLA-DR^+^CD56^bright^ subset is characterized by a lower maturation state compared to adaptive CD57^+^NKG2C^+^ cells, the intensive proliferation of CD57^−^ NK cells in donors with a high proportion of HLA-DR^+^CD56^bright^ cells may be driven by a mechanism distinct from the adaptive memory cell response.

Using the proportions of HLA-DR^+^, HLA-DR^+^CD56^bright^, KIR2DL2/3^+^, NKG2C^+^, and NKG2A^+^ populations as variables, we performed a cluster analysis of donors and identified three main clusters with distinct NK cell phenotypic properties and HLA genotype (Figure 1e and Figure S1c,d). The NK cell compartment of the smallest group, Cluster 1, was characterized by high proportions of more mature KIR^+^, CD57^+^, and NKG2C^+^ NK cells; cells with an adaptive phenotype (CD57^+^NKG2C^+^ and KIR^+^NKG2C^+^); as well as a high proportion of activated HLA-DR^+^ cells and a reduced proportion of NKG2A^+^ cells. Serum HCMV IgG antibody titers in these donors were higher compared to the other clusters (Figure 1f). Cluster 3 comprised donors with high proportions of HLA-DR^+^ and HLA-DR^+^CD56^bright^ cells and NKG2A^+^ cells, lower KIR and CD57 expression, and a trend toward a higher proportion of less differentiated CD56^bright^ cells. Donors in Cluster 3 had reduced HCMV IgG titers, with a trend toward lower NK cell counts in peripheral blood (Figure 1f and Figure S1c). Individuals in the most populous group, Cluster 2, occupied an intermediate position between Cluster 1 and Cluster 3 with respect to the expression levels of the maturation markers KIR, NKG2A, and CD57. Their NK cells were characterized by a reduced proportion of activated HLA-DR^+^ cells and intermediate HCMV IgG titers.

Clustering of ex vivo NK cell phenotype data identified several patterns in HCMV-associated marker expression around the donor NK cells, which implies their differences in maturation state and activation level. These patterns may affect NK cell responses to HCMV peptide presentation in vitro, including capacities for KIR^+^NKG2C^+^ cell accumulation.

### 2.2. Ex Vivo Phenotype Determines the Pattern of KIR^+^NKG2C^+^ NK Cell Enrichment in Response to LFL Peptide Stimulation

**Figure 2:**
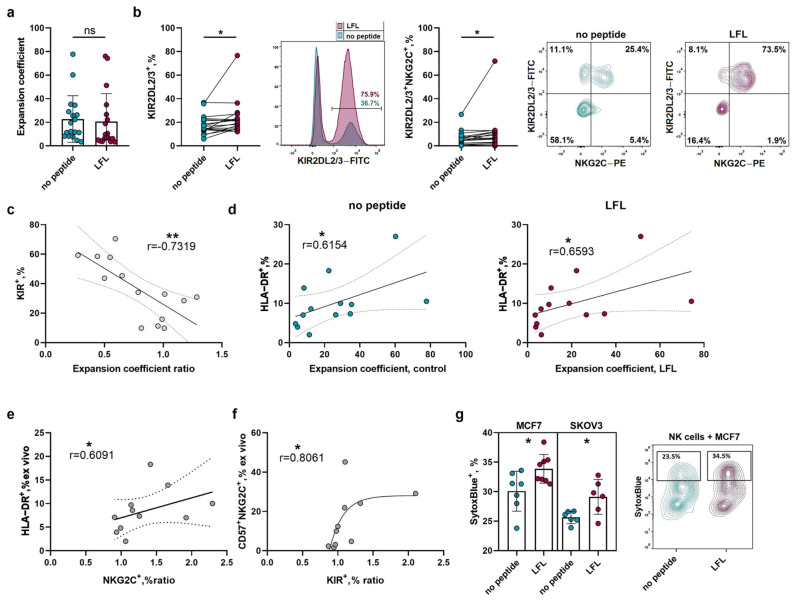
Proliferative activity and phenotype of bulk NK cell cultures obtained with and without LFL presentation: (**a**) expansion coefficient of bulk NK cell cultures after cultivation with and without HCMV peptide presentation, number of donors: N = 18 (HCMV^+^-13, HCMV^−^-5); (**b**) proportions of KIR^+^ and KIR^+^NKG2C^+^ cells in bulk NK cell cultures obtained with and without peptide presentation, with representative cytometric data, N = 18 (HCMV^+^-13, HCMV^−^-5); (**c**) correlation between the relative expansion coefficient K = K(LFL)/K (no peptide) and the proportion of KIR^+^ NK cells ex vivo, N = 14 (HCMV^+^-9, HCMV^−^-5); (**d**) correlation between the expansion coefficient of NK cell cultures obtained without (left) and with (right) LFL presentation and the proportion of HLA-DR^+^ NK cells ex vivo, N = 13 (HCMV^+^-8, HCMV^−^-5); (**e**) correlation between the relative proportion of NKG2C^+^ (NKG2C ratio = NKG2C^+^,% (LFL)/NKG2C^+^,% (no peptide)) cells in cultures obtained with LFL presentation and the proportion of HLA-DR^+^ ex vivo, N = 11 (HCMV^+^-6, HCMV^−^-5); (**f**) correlation between the relative proportion of KIR^+^ cells (KIR ratio = KIR^+^,% (LFL)/KIR^+^,% (no peptide)) in cultures presented with the LFL peptide and the proportion of CD57^+^NKG2C^+^ ex vivo, N = 11 (HCMV^+^-6, HCMV^−^-5); (**g**) proportion of non-viable cells in spheroids of HLA-E-expressing tumor lines MCF7 and SKOV3 after co-incubation with bulk cultures of NK cells activated with and without peptide presentation, with representative cytometric data; 6 to 8 replicates are presented. Cultivation time T = 14 days, total number of donors: N = 18 (HCMV^+^-13, HCMV^−^-5). Statistical analysis was performed using a nonparametric Wilcoxon’s test (**b**) or nonparametric Mann–Whitney test (**a**,**g**) (* *p* < 0.05, ** *p* <0.01, ns—not significant); means ± SD are shown. Correlation analysis was done using Spearman’s correlation for non-normally distributed data; *p* < 0.05 was considered statistically significant.

To investigate the relationship between ex vivo markers and the proliferative response of NK cells to HCMV peptide presentation, we established bulk NK cell cultures primed with the LFL peptide (LFL cultures) using a protocol described in our previous work [24]. Unstimulated cultures served as controls (control cultures). LFL presentation did not result in a generalized increase in NK cell proliferative activity (Figure 2a). However, LFL cultures exhibited significantly higher proportions of KIR^+^ and KIR^+^NKG2C^+^ cells compared to control cultures (Figure 2b). A higher relative expansion coefficient in LFL cultures was associated with a lower ex vivo frequency of KIR^+^ cells (Figure 2c). Notably, greater overall expansion was observed in NK cell cultures derived from donors with a higher proportion of HLA-DR^+^ cells, regardless of the stimulation condition (Figure 2d). LFL cultures from donors with a high frequency of HLA-DR^+^CD56^bright^ NK cells similarly proliferated more intensively, a trend mirrored in control cultures (Figure S2a). Taken together, NK cells from donors—characterized by high proportions of HLA-DR^+^ and HLA-DR^+^CD56^bright^ cells and a low proportion of KIR^+^ cells—with the features of Cluster 3 display the highest proliferative capacity. An elevated relative frequency of NKG2C^+^ cells in LFL cultures was observed in donors with a high ex vivo proportion of HLA-DR^+^ cells—a feature characteristic of Clusters 1 and 3 (Figure 2e). NK cells from donors with a higher proportion of adaptive CD57^+^NKG2C^+^ cells, as seen in Cluster 1, showed a greater relative enrichment of KIR^+^ cells upon LFL peptide stimulation (Figure 2f).

We further assessed the antitumor activity of the resulting cultures using a model system based on 3D spheroids of MCF7 and SKOV3 tumor cell lines, both of which express HLA-E (Figure S2b) and carry the HLA-C2/C2 genotype. Following co-incubation with LFL NK cell cultures, spheroids of both types exhibited a significantly higher proportion of non-viable SytoxBlue^+^ cells compared to spheroids incubated with control cultures (Figure 2g). We also evaluated the degranulation capacity of KIR^+^ cells within the cultures and demonstrated that cells co-expressing KIRs and NKG2C degranulate more actively in the presence of HLA-E-expressing MCF7 and SKOV3 spheroids compared to the NKG2C-negative fraction (Figure S2c). Degranulation was accompanied by decreasing NKG2C expression, which indicates NKG2C internalization upon target recognition (Figure S2d).

Collectively, these findings establish LFL presentation in vitro as an effective strategy for expanding KIR^+^NKG2C^+^ NK cells with enhanced cytotoxic potential against HLA-E-expressing solid tumors. However, NK cells from donors with different ex vivo characteristics display distinct response patterns upon in vitro stimulation.

### 2.3. Enhanced Proliferation of KIR^+^NKG2C^+^ Cells in Response to LFL Peptide Presentation Is Associated with High Levels of KIR, NKG2C, and NKG2A in NK Cells Ex Vivo

The accumulation of KIR^+^NKG2C^+^ cells can be driven either by the proliferation of pre-existing KIR^+^NKG2C^+^ cells or by de novo acquisition of KIR and NKG2C expression in initially receptor-negative subsets. To determine which of these mechanisms predominates upon LFL peptide presentation, we performed cell sorting to establish cultures of 100 NK cells from the following subpopulations: CD57^−^KIR^+/−^NKG2C^+/−^ and CD57^+^KIR^+^NKG2C^+/−^ (Figure S3). Subsequently, these were activated in the presence of feeder cells with or without LFL.

**Figure 3:**
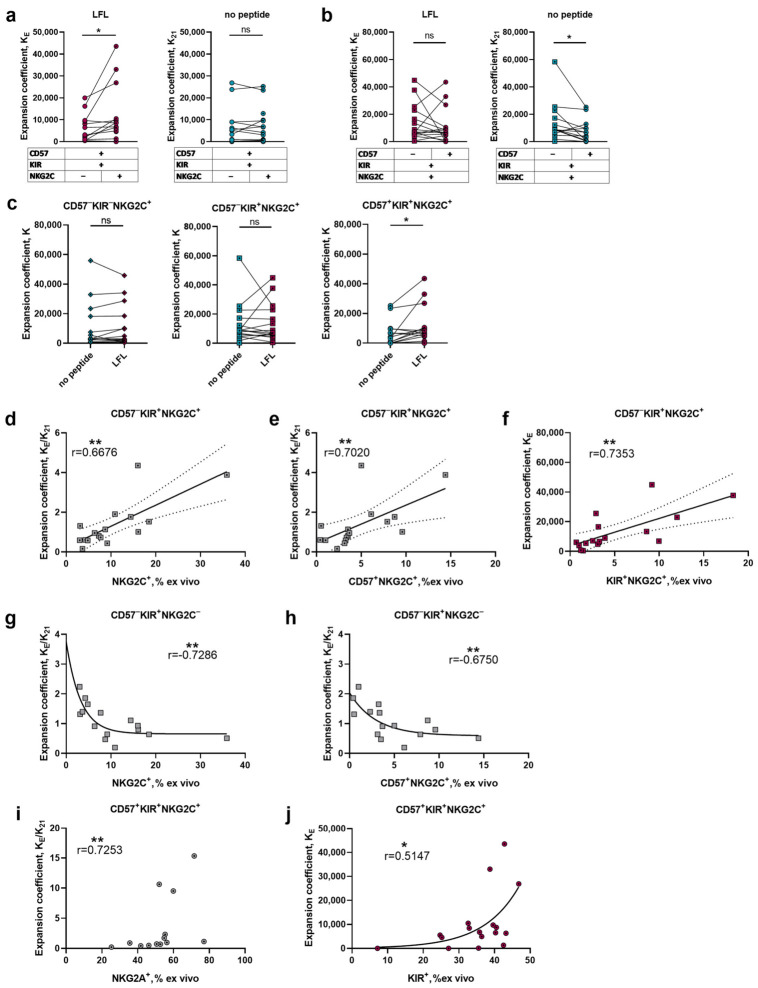
Proliferative activity of NK cell subsets with different expression of KIR, NKG2C, and CD57 cultured with or without LFL presentation: (**a**) expansion coefficient of CD57^+^KIR^+^ cultures with different NKG2C expression, which were (left) and were not (right) presented with LFL peptide; (**b**) expansion coefficient of KIR^+^NKG2C^+^ cultures with different CD57 expression, which were (left) and were not (right) presented with LFL peptide; (**c**) expansion coefficient of NKG2C^+^ cultures with different KIR and CD57 expression, which were cultured with or without LFL peptide presentation; (**d,e**) correlation between the relative expansion ratio upon presentation of LFL peptide to CD57-KIR+NKG2C+ cells and the proportions of NK cells NKG2C^+^ (**d**) and CD57^+^NKG2C^+^ (**e**) ex vivo; (**f**) correlation between the expansion ratio upon presentation of LFL peptide to CD57^−^KIR^+^NKG2C^+^ cells and the proportion of KIR^+^NKG2C^+^ NK cells ex vivo; (**g**,**h**) correlation between the relative expansion ratio upon presentation of LFL peptide to CD57^−^KIR^+^NKG2C^−^ cells and the proportions of NK cells NKG2C^+^ (**g**) and CD57^+^NKG2C^+^ (**h**) ex vivo; (**i**) correlation between the relative expansion coefficient upon presentation of LFL peptide to CD57^+^KIR^+^NKG2C^+^ cells and the proportion of NK cells NKG2A^+^ ex vivo; (**j**) correlation between the expansion coefficient upon presentation of LFL peptide to CD57^+^KIR^+^NKG2C^+^ cells and the proportion of NK cells KIR^+^ ex vivo. Duration of cultivation: T = 12 days, number of donors: N = 17 (HCMV^+^-11, HCMV^−^-6). Statistical analysis was performed using a nonparametric Wilcoxon’s test (**a**–**c**) (* *p* < 0.05, ** *p* < 0.01, ns—not significant). Correlation analysis was done using Pearson’s or Spearman’s correlation; *p* < 0.05 was considered statistically significant.

We first assessed the effect of LFL on the proliferative capacity of each subset. CD57^+^KIR^+^NKG2C^+^ cells with an adaptive phenotype displayed greater proliferative activity upon LFL presentation compared to CD57^+^KIR^+^NKG2C^−^ cultures, while their expansion rate was comparable to that of the less differentiated CD57^−^KIR^+^NKG2C^+^ adaptive cells (Figure 3a,b). No such differences were observed under peptide-free culture conditions. The CD57^+^KIR^+^NKG2C^+^ subset mounted more pronounced proliferative responses upon LFL presentation compared to the control without peptide (Figure 3c and Figure S4a). These findings indicate that an LFL-specific proliferative response is most pronounced in NK cell subsets co-expressing KIRs and NKG2C.

We found a moderate relationship between a higher expansion rate of the CD57^−^KIR^+^NKG2C^+^ subset in response to LFL (relative to expansion without peptide; K ratio) and greater ex vivo proportions of NKG2C^+^ and CD57^+^NKG2C^+^ cells (Figure 3d,e). NK cells from donors with a higher ex vivo frequency of KIR^+^NKG2C^+^ cells proliferated more vigorously upon LFL stimulation, but not in the control cultures (Figure 3f and Figure S4b). In contrast, the CD57^−^KIR^+^ less mature subset lacking NKG2C demonstrated a moderate negative correlation between the K ratio and ex vivo proportions of NKG2C^+^ and CD57^+^NKG2C^+^ cells (Figure 3g,h). The relative expansion rate of adaptive CD57^+^KIR^+^NKG2C^+^ NK cells in response to LFL presentation (K ratio) positively correlated with the ex vivo proportion of NKG2A^+^ cells (Figure 3i). Furthermore, enhanced proliferative activity of these cells upon LFL stimulation, but not in control cultures, was observed in NK cells obtained from donors with a higher ex vivo frequency of KIR^+^ cells (Figure 3j and Figure S4c).

Taken together, LFL-driven accumulation of both adaptive CD57^+^KIR^+^NKG2C^+^ cells and their potential progenitors CD57^−^KIR^+^NKG2C^+^ is most pronounced in NK cell cultures from donors with Cluster 1 characteristics. Conversely, CD57^−^KIR^+^NKG2C^−^ cells from such donors display attenuated LFL-associated proliferation. Notably, adaptive CD57^+^KIR^+^NKG2C^+^ cells mounted an LFL-dependent proliferative response that was associated with a high ex vivo NKG2A expression level—a feature characteristic of Cluster 3 donors. These findings may indirectly point to a distinct role for the NKG2A receptor in shaping the immune response against HCMV.

### 2.4. De Novo NKG2C Expression in KIR^+^ NK Cell Cultures Is Associated with a High Ex Vivo Proportion of HLA-DR^+^ Cells

**Figure 4:**
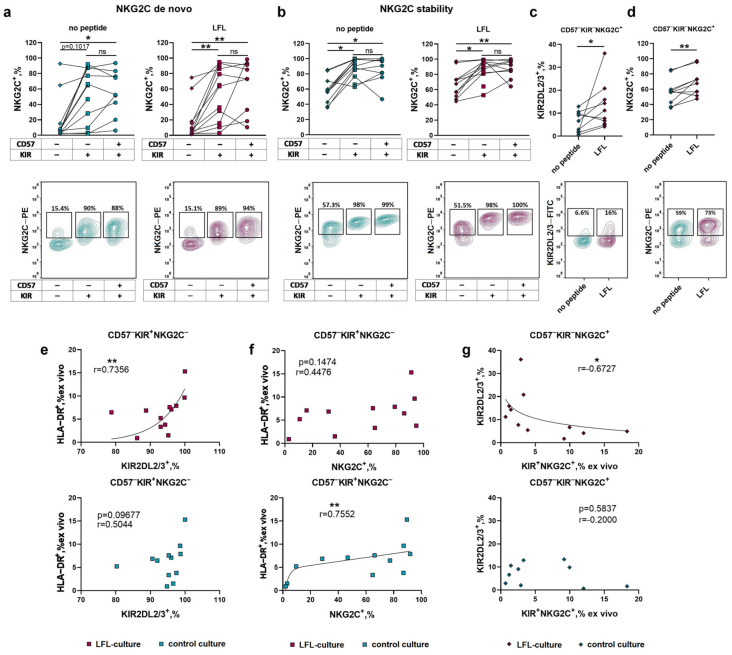
Phenotype of NK cell subset cultures with different KIR, NKG2C, and CD57 expression profiles, activated in the presence or absence of LFL peptide: (**a**) De novo NKG2C expression in cultures of NKG2C-negative subsets with different expression of KIR and CD57, activated in the presence or absence of LFL peptide; (**b**) stability of NKG2C expression in cultures of NKG2C-positive subsets with different expression of KIR and CD57, activated in the presence or absence of LFL peptide; (**c**) de novo KIR expression in cultures of CD57^−^KIR^−^NKG2C^+^ subset, activated in the presence or absence of LFL; (**d**) stability of NKG2C expression in cultures of CD57^−^KIR^−^NKG2C^+^, activated in the presence or absence of LFL; (**e**,**f**) correlation between the proportion of HLA-DR^+^ NK cells ex vivo and the stability of KIR expression (**e**) and NKG2C de novo expression (**f**) in CD57^−^KIR^+^NKG2C^−^ subset cultures that were and were not presented with LFL; (**g**) correlation between the proportion of KIR^+^NKG2C^+^ NK cells ex vivo and KIR de novo expression in CD57^−^KIR^−^NKG2C^+^ subset cultures that were and were not presented with LFL. Duration of cultivation: T = 12 days, number of donors: N = 17 (HCMV^+^-11, HCMV^−^-6). Statistical analysis was performed using a nonparametric Friedman’s test followed by Dunn’s multiple comparison post hoc test (**a**,**b**) or Wilcoxon’s test (**c**,**d**) (* *p* < 0.05, ** *p* < 0.01, ns—not significant). Correlation analysis was done using Spearman’s correlation; *p* < 0.05 was considered statistically significant.

KIR^+^NKG2C^+^ cell accumulation may also arise through de novo acquisition of KIR and/or NKG2C expression. LFL cultures of KIR-positive subsets consistently showed higher proportions of de novo NKG2C^+^ cells compared to KIR-negative cultures; the same trend was observed in the control cultures (Figure 4a). The NKG2C receptor expression was also more stable in the KIR^+^NKG2C^+^ subset-derived cultures compared to KIR-negative cultures, regardless of CD57 and stimulation type (Figure 4b). We did not detect differences in KIR stability between KIR-positive subset cultures that co-expressed CD57 or NKG2C or were differently stimulated (Figure S5a). De novo KIR expression in CD57-negative subsets was independent of NKG2C expression (Figure S5d). LFL-associated phenotypic differences in KIR or NKG2C levels were detected exclusively in the least differentiated NKG2C-positive subset—CD57^−^KIR^−^NKG2C^+^. Peptide-stimulated cultures of this subset exhibited a higher proportion of de novo KIR^+^ cells and more stable NKG2C expression compared to control cultures (Figure 4c,d). In all other subpopulations, the stability and de novo acquisition of KIRs and NKG2C on the cell surface were independent of peptide presentation (Figure S5c–f).

We next examined the relationship between the ex vivo NK cell phenotype and the proportions of KIR^+^ and NKG2C^+^ cells in the resulting cultures. Regarding CD57^−^KIR^+^NKG2C^−^ cultures, KIR expression stability positively correlated with an ex vivo proportion of HLA-DR^+^ NK cells in LFL-stimulated samples (Figure 4e). De novo NKG2C^+^ acquisition was similarly associated with the ex vivo proportion of HLA-DR^+^ NK cells, although only in the control samples (Figure 4f). Within the CD57^−^KIR^−^NKG2C^+^ subset, a higher proportion of de novo KIR^+^ cells in peptide-stimulated cultures was moderately associated with a lower ex vivo frequency of KIR^+^NKG2C^+^ cells (Figure 4g). No analogous association was observed in control cultures.

Taken together, a high stability of activating NKG2C receptor expression and a high rate of de novo NKG2C acquisition are features of KIR-expressing subpopulations. Notably, within the CD57^−^KIR^+^NKG2C^−^ subset, a high proportion of de novo NKG2C^+^ cells is associated with a high ex vivo frequency of HLA-DR^+^ cells—a phenotypic profile characteristic of NK cells from Cluster 1 and Cluster 3 donors. KIR expression stability was independent of NKG2C co-expression; however, enhanced de novo KIR^+^ cell acquisition relative to control conditions can occur upon HCMV peptide presentation in the less differentiated CD57^−^KIR^−^NKG2C^+^ cells. The lowest level of de novo KIR expression in this subset was observed in NK cell cultures derived from donors whose ex vivo phenotype is characteristic of Cluster 1.

### 2.5. High Functional Activity of KIR^+^NKG2C^+^ NK Cells Is Associated with Increased Ex Vivo NKG2A Levels

We assessed the functional activity of the subset cultures by measuring cytokine-dependent IFNγ production and degranulation in response to co-incubation with K562 cells, which lack HLA class I surface expression.

**Figure 5:**
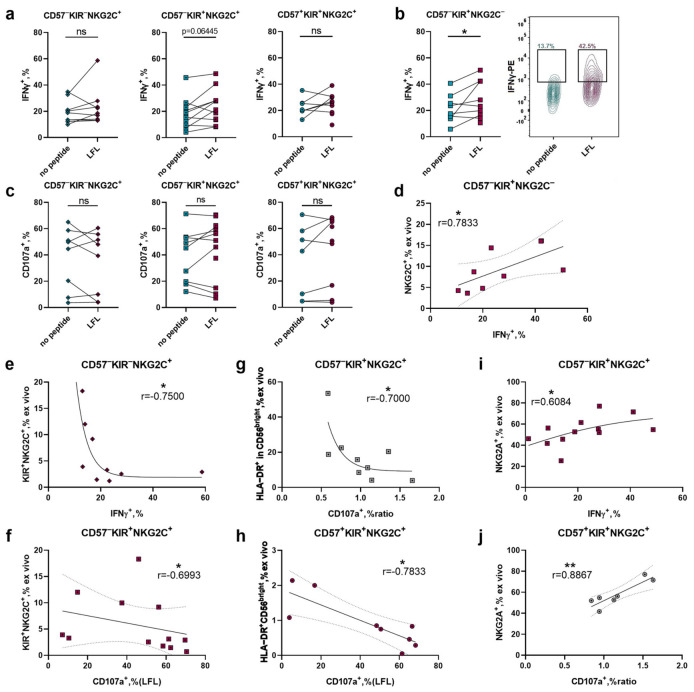
Functional activity of NK cell subset cultures with different expression of KIR, NKG2C, and CD57, activated in the presence or absence of LFL peptide: (**a**) proportion of IFNγ-producing NK cells in cultures of NKG2C-positive subsets with different expression of KIR and CD57; (**b**) proportion of IFNγ-producing NK cells in cultures of the CD57^−^KIR^+^NKG2C^−^ subset, to which LFL peptide was and was not presented; (**c**) proportion of degranulating CD107a^+^ NK cells in cultures of NKG2C-positive subsets with different expression of KIR and CD57 upon co-incubation with K562 targets; (**d**) correlation between the proportion of IFNγ-producing cells in CD57^−^KIR^+^NKG2C^−^ subset cultures presented with LFL peptide and the proportion of NK cells NKG2C^+^ ex vivo; (**e**) correlation between the proportion of IFNγ-producing cells in CD57^−^KIR^−^NKG2C^+^ subset cultures presented with LFL peptide and the proportion of NK cells KIR^+^NKG2C^+^ ex vivo; (**f**) correlation between the proportion of IFNγ-producing cells in CD57^−^KIR^+^NKG2C^+^ subset cultures presented with LFL peptide and the proportion of NK cells KIR^+^NKG2C^+^ ex vivo; (**g**) correlation between the proportion of CD107a^+^ cells in CD57^−^KIR^+^NKG2C^+^ subset cultures presented with LFL peptide and the proportion of HLA-DR^+^ NK cells in the CD56^bright^ fraction ex vivo; (**h**) correlation between the relative proportion of CD107a^+^ cells in CD57^+^KIR^+^NKG2C^+^ subset cultures presented with LFL peptide and the proportion of HLA-DR^+^CD56^bright^ NK cells ex vivo; (**i**) correlation between the proportion of IFNγ-producing cells in CD57^−^KIR^+^NKG2C^+^ subset cultures presented with LFL peptide and the proportion of NKG2A^+^ NK cells ex vivo; (**j**) correlation between the proportion of CD107a^+^ cells in CD57^+^KIR^+^NKG2C^+^ subset cultures presented with the LFL peptide and the proportion of NKG2A^+^ NK cells ex vivo. Duration of cultivation: T = 17 days, number of donors: N = 17 (HCMV^+^-11, HCMV^−^-6). Statistical analysis was performed using a nonparametric Wilcoxon’s test (**a**–**c**) (* *p* < 0.05, ** *p* < 0.01, ns—not significant). Correlation analysis was done using Spearman’s correlation; *p* < 0.05 was considered statistically significant.

Compared to control cultures, LFL-stimulated CD57^−^KIR^+^ cultures displayed significantly higher IFNγ production in the NKG2C-negative fraction, with a similar trend observed in the NKG2C-expressing fraction (Figure 5a,b). In all other subsets, the LFL peptide presentation had no significant effect on IFN-γ production (Figure 5a and Figure S6a). No differences in the proportion of CD107a^+^ cells between LFL and control cultures were detected in any of the subsets (Figure 5c and Figure S6b).

We further investigated the relationship between the ex vivo NK cell phenotype and functional activity across cultures from different subsets. Enhanced IFN-γ production in LFL-stimulated CD57^−^KIR^+^NKG2C^−^ cultures was associated with a higher ex vivo proportion of NKG2C^+^ cells (Figure 5d and Figure S6c). A high ex vivo frequency of KIR^+^NKG2C^+^ cells was associated with reduced IFN-γ production in LFL-stimulated CD57^−^KIR^−^NKG2C^+^ cultures (Figure 5e and Figure S6d) and with attenuated cytotoxicity in LFL-stimulated CD57^−^KIR^+^NKG2C^+^ cultures (Figure 5f and Figure S6e). Reduced cytotoxicity of KIR^+^NKG2C^+^ cultures was also associated with a high ex vivo proportion of HLA-DR^+^ cells. Within the less mature CD57^−^KIR^+^NKG2C^+^ subset, a lower degranulation degree was observed in LFL cultures derived from donors with a higher proportion of HLA-DR^+^ cells within the CD56^bright^ compartment (Figure 5g). A higher ex vivo frequency of HLA-DR^+^CD56^bright^ cells was similarly associated with reduced degranulation activity in adaptive CD57^+^KIR^+^NKG2C^+^ cultures, regardless of stimulation condition (Figure 5h and Figure S6f). Analogous trends were observed across other NKG2C-expressing subsets (Figure 5h and Figure S6g–j).

Notably, a high proportion of NKG2A^+^ cells ex vivo was associated with more pronounced IFNγ production by CD57^−^KIR^+^NKG2C^+^ LFL cultures (Figure 5i and Figure S6k) and a higher proportion of CD107a^+^ cells, and, consequently, more pronounced cytotoxicity in CD57^+^KIR^+^NKG2C^+^ LFL cultures (Figure 5f). These results once again emphasize the special role of the NKG2A receptor, not only in the formation of the adaptive NK cell population, but also in the development of their functional profile.

Ex vivo phenotyping could be a tool for determining the intensity of proliferative response, phenotypic variability of NK cell cultures, and also their functional characteristics. High IFNγ production by CD57^−^KIR^+^NKG2C^−^ LFL cultures was associated with a high proportion of NKG2C^+^—characteristic of Cluster 1 individuals. At the same time, the ex vivo characteristics of Cluster 3 had conflicting prognostic properties. High proportions of the HLA-DR^+^CD56^bright^ and HLA-DR^+^ fractions in the CD56^bright^ population were associated with reduced cytotoxicity of NKG2C^+^ cultures. At the same time, a high proportion of NKG2A^+^ ex vivo was associated with more pronounced functional activity of KIR^+^NKG2C^+^ cultures presented with the HCMV peptide.

### 2.6. LFL-Stimulated KIR^+^NKG2C^+^ NK Cells Exhibited Enhanced Cytotoxic Activity Against HLA-E^+^ Tumor Spheroids

**Figure 6:**
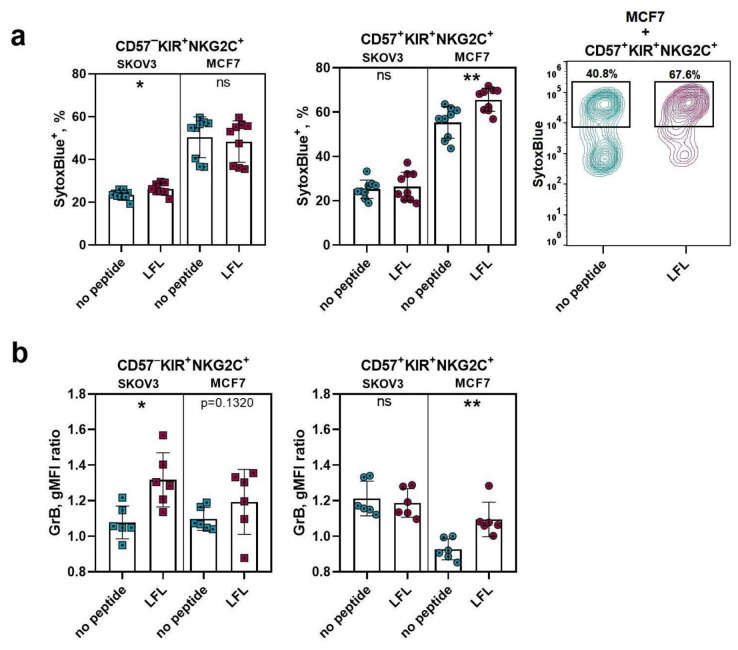
Cytotoxic activity of KIR^+^2C^+^ NK cell cultures against HLA-E^+^ tumors: (**a**) the proportion of non-viable SytoxBlue^+^ cells in spheroids of HLA-E-expressing tumor lines MCF7 and SKOV3 after co-incubation with KIR^+^NKG2C^+^ NK cells cultures, which were activated with and without LFL presentation; (**b**) the level of GrB penetration in spheroids of HLA-E-expressing tumor lines MCF7 and SKOV3 after co-incubation with KIR^+^NKG2C^+^ NK cells cultures, which were activated with and without LFL presentation. Number of replicates: N = 9 (**a**) or N = 6 (**b**). Statistical analysis was performed using a nonparametric Mann–Whitney test (* *p* < 0.05, ** *p* < 0.01, ns—not significant).

We also assessed the cytotoxic activity of NK cells co-expressing KIRs and NKG2C against HLA-E^+^ tumor lines MCF7 and SKOV3 spheroids. Target cell viability in spheroids was assessed by the proportion of cells unstained with SytoxBlue, and by the level of granzyme B (GrB) penetration. LFL cultures of the CD57^−^KIR^+^NKG2C^+^ subset eliminated SKOV3 targets, but not MCF7, with greater efficiency than control cultures: a higher proportion of SytoxBlue^+^ dying cells and a higher level of GrB expression were detected in SKOV3 spheroids (Figure 6a,b). Cells with a more differentiated phenotype, CD57^+^KIR^+^NKG2C^+^, when stimulated with the LFL peptide, exhibited greater cytotoxicity compared to control cultures against MCF7 spheroids, but not SKOV3 spheroids: MCF7 cells also exhibited a higher proportion of SytoxBlue^+^ cells and a higher GrB content (Figure 6a,b). Thus, NK cells co-expressing KIRs and NKG2C exhibit varying efficacy against solid tumors depending on their differentiation state.

## 3. Discussion

The NK cells from different donors exhibit extensive functional and phenotypic diversity, which is shaped by individual donor characteristics, including the history of encounters with pathogens. HCMV infection modulates the NK cell population composition by stimulating the accumulation of NKG2C^+^ cells capable of recognizing HLA-E-expressing targets. In HCMV-seropositive donors, in comparison with HCMV-seronegative ones, we identified a higher proportion of activated HLA-DR^+^ cells, as well as cells co-expressing the inhibitory KIRs and NKG2C. Expression of KIR family receptors for self-HLA-I molecules is characteristic of cells with pronounced cytotoxic potential and a high content of lytic granules [14]. However, during infection, NK cells are capable of not only eliminating infected cells, but also acting as regulators and even antigen presenters. It was reported that NK cells present cytomegalovirus peptides within HLA-DR to T cells, engaging the adaptive immune system in the immune response [25,26,27]. Thus, HCMV infection can be restrained through cellular elements of both innate and adaptive immunity, and a predisposition to one of these antiviral tactics may be associated with marker expression by the NK cells ex vivo.

We hypothesized that donor NK cells with different patterns of HCMV-associated molecule expression would respond to in vitro stimulation, including LFL peptide presentation, with varying intensity. We conducted a cluster analysis of 77 individuals with HLA-C1/C1 and HLA-C2/C1 genotypes based on the expression of HCMV-modulated markers KIR2DL2/3, NKG2C, NKG2A, and HLA-DR, as well as the proportion of HLA-DR^+^CD56^bright^ cells, a high level of which was previously shown to be associated with intense expansion of the CD57-negative fraction in the presence of LFL. Individuals were divided into three clusters with distinct characteristics.

Cluster 1 individuals were characterized by high anti-HCMV antibody titers and the largest proportions of adaptive NK cells expressing NKG2C, CD57, KIR, and low NKG2A expression. An increase in cytotoxic NK effectors in the bloodstream of donors in Cluster 1 suggests a predisposition to an effector role for NK cells in the HCMV-directed response. Cluster 3 included individuals with lower anti-HCMV antibody titers, high levels of CD56^bright^ and NKG2A^+^ cells, and reduced expression of KIR, CD57, and NKG2C—indicating low NK cell maturity. Additionally, Cluster 3 was characterized by a lower NK cell count in the bloodstream compared to other clusters, which might indicate migration of mature NK cells into tissues. Cluster 2 individuals occupied an intermediate position between Cluster 1 and Cluster 3 in terms of NK cell maturity marker expression, such as KIR, NKG2A, and CD57, and had a reduced proportion of activated HLA-DR^+^ NK cells. Notably, each of the clusters showed an elevated subset corresponding to one of the three NK cell cohorts recently identified based on transcriptome analysis [28]. Cluster 1 had elevated levels of cells with the adaptive phenotype, which are classified as NK3 cells. The NK cells of individuals in Cluster 3 showed elevated levels of cells with characteristics of the NK2 subset. Cluster 2 was characterized by intermediate expression of maturity markers, similar to cells of the NK1 subset.

A number of studies have shown K562-based feeder cells expressing membrane-bound IL-21 to be a perspective stimulus for therapeutic NK cell accumulation [29,30,31,32]. We also previously characterized K562-mbIL21 feeder cells as an effective tool for NK cell accumulation [33] and manufactured HLA-E-expressing feeder cells on their basis [24].

HCMV peptide presentation in the context of HLA-E in vitro led to the accumulation of KIR^+^NKG2C^+^ NK cells in bulk cultures. We previously demonstrated that NKG2C-positive clonal NK cell cultures exhibited the most stable KIR expression during in vitro NK cell expansion [34]. It is also known that HCMV infection is accompanied by clonal or polyclonal expansion of NKG2C^+^ cells co-expressing the inhibitory receptor KIRs specific to self-HLA-I molecules [16]. NK cell reactivation in response to HCMV after allogeneic bone marrow transplantation is also followed by polyclonal expansion of cells co-expressing KIRs and NKG2C [35]. Thus, by using feeder cells presenting the HCMV peptide (LFL) within HLA-E, it is possible to mimic physiological conditions in vitro and induce the accumulation of cells bearing the desired phenotype.

The LFL-derived expansion intensity and phenotypic changes in NK cell cultures were associated with the NK cell phenotype ex vivo. A more pronounced proliferative response of bulk NK cells to LFL was associated with a reduced content of KIR^+^ cells and high proportions of activated HLA-DR^+^ and HLA-DR^+^CD56^bright^ cells ex vivo. KIR-positive cells are characterized by a higher degree of differentiation and reduced expression of cytokine receptors. In particular, more differentiated NK cells typically express IL-2Rβ, a subunit of the IL-2 receptor with lower affinity compared to IL-2Rα [36]. This explains the reduced accumulation of fractions with high KIR^+^ content. We previously demonstrated that intense expansion of CD57-negative NK cells upon LFL presentation is associated with a high proportion of HLA-DR^+^CD56^bright^ cells [24]. HLA-DR^+^ cells in the CD56^bright^ population are known to differ from HLA-DR-negative ones by their high expression of genes associated with proliferative activity and apoptosis resistance (BIRC5), as well as by their greater mitochondrial mass [37]. This explains the intensive in vitro expansion of NK cells obtained from donors with a high HLA-DR^+^CD56^bright^ cell proportion.

**Figure 7:**
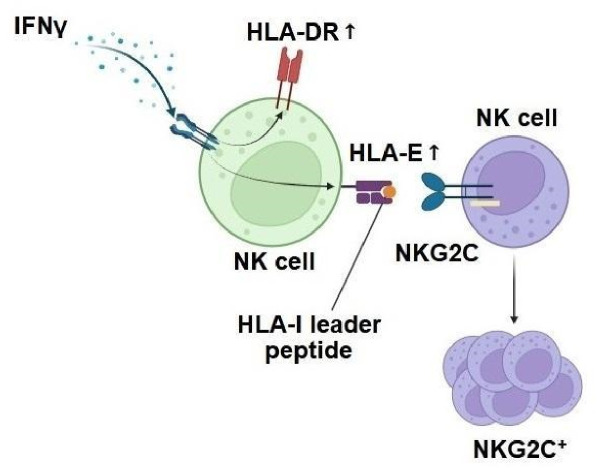
Plausible mechanism of NKG2C^+^ NK cell expansion upon IFNγ stimulation. The ↑ arrow indicates an increase in marker expression.

We showed that a high level of HLA-DR^+^ cells ex vivo is associated with more intense accumulation of NKG2C^+^ NK cells upon LFL presentation, while LFL-associated accumulation of KIR^+^ cells is associated with a high content of adaptive CD57^+^NKG2C^+^ cells ex vivo. Accumulation of NK cells co-expressing KIRs and NKG2C upon LFL stimulation can be accomplished in two ways: through expansion of NK cells of the KIR^+^NKG2C^+^ fraction, or through de novo expression of KIRs and NKG2C in negative fractions. To clarify which of these mechanisms dominates in response to LFL, we obtained NK cell cultures from subsets differing in the levels of KIR, NKG2C, and the maturity marker CD57, under stimulation in the presence or absence of the LFL peptide. De novo NKG2C expression was observed in the CD57^−^KIR^+^NKG2C^−^ subset, and a higher proportion of NKG2C^+^ was associated with a high content of HLA-DR^+^ ex vivo. In NK cells, HLA-DR expression is closely linked to IFNγ production, which autocrinally promotes increased expression of this marker [37]. In addition, Basal HLA-E level is characteristic of B, T, and NK cells [38], and IFNγ is able to induce increased HLA-E expression [39,40]. In the case of NKG2C de novo appearance on the cell surface, high HLA-E expression on surrounding NK cells can potentially provide increased binding avidity, a more pronounced activating intracellular signal from NKG2C receptors, and active proliferation. However, it is important to note that native HLA-E molecules usually express when being loaded by the HLA-I leader peptide (LP). HLA-E+LP complexes are inferior to the HLA-E+LFL complex in terms of their stimulatory effect on NKG2C^+^ NK cells [21]. Thus, HLA-DR and the NKG2C ligand, HLA-E, share the IFNγ stimulus, and an increased proportion of HLA-DR^+^ NK cells ex vivo may be a marker of IFNγ-mediated changes in the NK cell phenotype, indirectly facilitating the accumulation of NKG2C-positive cells (Figure 7). Increased HLA-E expression in vitro induces proliferative selection of cells that have begun to express NKG2C [39]. This may explain the LFL-associated accumulation of NKG2C^+^ NK cells in vitro and in vivo. However, the mechanisms underlying the initiation of de novo NKG2C expression remain to be elucidated.

Notably, CD57^−^KIR^+^NKG2C^−^ cell LFL cultures exhibited increased cytokine-induced IFNγ production compared to the corresponding control cultures. These cultures did not exhibit an increased proportion of NKG2C^+^ NK cells; however, additional presentation of the HCMV peptide by feeder cells could have led to modification of the IFNG locus, characteristic of the adaptive state of NK cells [41]. A similar trend was observed for cells of the CD57^−^KIR^+^NKG2C^+^ subset, but not for the more differentiated CD57^+^KIR^+^ fractions. This may be associated with reduced expression of IL-12 and IL-18 cytokine receptors in the CD57^+^ NK cell population [28].

Several studies have shown that in vitro presentation of HCMV peptides leads to intense proliferation of NKG2C-positive NK cells [22], including those expressing KIRs [23]. In our study, we clarified that LFL-dependent proliferation is observed specifically in cultures of highly differentiated NK cells with the adaptive phenotype CD57^+^KIR^+^NKG2C^+^. A specific response was observed even after a single stimulation with the HLA-E+LFL complex. Less differentiated CD57^−^KIR^+^NKG2C^+^ NK cells, unlike CD57^+^KIR^+^NKG2C^+^, did not respond to peptide presentation with increased proliferation; however, a higher expansion coefficient upon LFL presentation was associated with a high content of adaptive NK cells ex vivo.

The expansion rate of CD57^+^KIR^+^NKG2C^+^ adaptive NK cells in LFL cultures did not exceed the expansion rates of less differentiated CD57-negative cultures. One approach to enhancing LFL-mediated proliferative activity is co-stimulation of NKG2C^+^ NK cells with HLA-E and the inflammatory interleukins IL-12 and IL-18 [22,42]. Several studies have shown that IL-12 stimulation leads to an increase in the NKG2A^+^ cell proportion in the bloodstream [43] and promotes transient expression of NKG2A in NKG2C^+^ NK cells [42,44]. As NKG2A is a checkpoint molecule, such phenotype changes may affect NK cell cytotoxic potential. However, we previously showed that cells co-expressing NKG2C and NKG2A exhibit high proliferative potential, increased expression of cytokine receptors and intercellular adhesion molecules, while not differing in the expression of adaptive state markers and maintaining a high level of antibody-dependent cytotoxicity [45]. Notably, LFL presentation to NKG2C^+^NKG2A^+^ NK cells did not suppress their expansion in vitro. In this study, we found that a high content of NKG2A^+^ NK cells ex vivo is associated with a more pronounced LFL-mediated expansion of adaptive CD57^+^KIR^+^NKG2C^+^ cells and increased cytokine-mediated functional activity of KIR^+^NKG2C^+^ cells: CD57-negative cells exhibited increased IFNγ production, while CD57^+^ cells degranulated more actively when co-incubated with K562. Thus, increased levels of NKG2A-positive cells may be associated with prominent sensitivity of NK cells to cytokine stimulation, and, as a consequence, more pronounced proliferation in vitro.

Tumor microenvironments often exhibit hypoxia and elevated concentrations of immunosuppressive molecules, particularly IL-10. Increased IL-10 expression and oxygen deprivation are known to induce tissue expression of the non-classical HLA molecule, HLA-G [46,47,48]. The HLA-G leader sequence, identical to one of the cytomegalovirus peptides, VMAPRTLFL (LFL), is presented as part of HLA-E on the surface of tumor cells. Many tumors evade immune surveillance by expressing HLA-E and suppressing NK cell reactivity via the NKG2A receptor. Functionally active NK cells producing IFNγ may contribute to increased HLA-E expression in tumors and their resistance, as has been demonstrated for multiple myeloma, AML, ovarian cancer, and colorectal cancer [49,50,51]. Therefore, LFL-mediated NK cell activation and accumulation of NKG2C-expressing effectors are of particular interest in the context of overcoming the NKG2A-HLA-E checkpoint. We assessed the cytotoxic activity of cultures presented with the LFL peptide against spheroids of HLA-E-expressing tumor cultures. Both NK cells from bulk cultures and cells from KIR+NKG2C+ subset cultures induced increased cell death in SKOV3 and MCF7 spheroids. Moreover, more differentiated CD57^+^KIR^+^NKG2C^+^ cells exhibited greater cytotoxicity against MCF7, while CD57^−^KIR^+^NKG2C^+^ cells exhibited greater cytotoxicity against SKOV3. This may be due to the fact that other receptor–ligand axes, in addition to NKG2C-HLA-E, are also involved in spheroid elimination. We previously demonstrated that SKOV3 cells exhibit high expression of NKG2D receptor ligands, while MCF7 cells exhibit high expression of CX3CL1 [52]. NKG2D expression is characteristic of less differentiated NK2 cells and decreases with differentiation. CX3CR1 expression is observed in the more differentiated NK cell fraction [28].

In this study, we demonstrated that in vitro presentation of HCMV peptide to NK cells leads to the accumulation of cells co-expressing KIRs and NKG2C, which have enhanced cytotoxic potential against HLA-E-positive tumor spheroids. The type of accumulation was associated with the ex vivo expression of NK cell markers associated with HCMV infection. Active proliferation of CD57^+^KIR^+^NKG2C^+^ adaptive NK cells upon LFL presentation was associated with a high ex vivo NKG2A^+^ content; less differentiated CD57^−^KIR^+^NKG2C^+^ NK cells responded to peptide presentation with greater expansion when they were obtained from an individual with a high circulating adaptive cell content. Increased de novo NKG2C expression was associated with a high proportion of activated HLA-DR^+^ NK cells ex vivo. Thus, we identified the characteristics of individuals whose NK cells have the greatest potential to accumulate a fraction of HLA-E-targeted effectors co-expressing KIRs and NKG2C. Furthermore, we expanded our understanding of the NK cell response to HCMV peptide presentation.

## 4. Materials and Methods

### 4.1. Cell Lines

K562 and two K562-based modified feeder cell lines were used in this study: gene-modified K562 cells expressing membrane-bound IL-21 (K562-mbIL21); and HLA-E-modified K562-mbIL21 cells (K562-21E). For functional tests, ovarian cancer SKOV3 and breast cancer MCF7 cell lines were used. K562, SKOV3, and MCF7 cell lines were acquired from ATCC (Manassass, VA, USA); K562-mbIL21 cells were kindly provided by Dr. Dean Lee (MD Anderson Cancer Center, Houston, TX, USA); and K562-21E cells were obtained previously [24]. All mentioned cell lines were cultivated in complete RPMI-1640 medium (PanEco, Moscow, Russia) with 10% FBS (HyClone Laboratories, Logan, UT, USA), 2mM L-alanyl-glutamine (PanEco), 1% sodium pyruvate (PanEco), and 1% antibiotic–antimycotic (Millipore Sigma, St. Louis, MO, USA). Prior to experiments, feeder cells K562-mbIL21 and K562-21E were irradiated with 100 Gray and frozen. Then, both types of feeder cells were stimulated with the VMAPRTLFL peptide (LFL) at a concentration of 300 μM in serum-free Opti-MEM medium (Thermo Fisher Scientific, Waltham, MA, USA) for 5 h at 37 °C in 5% CO_2_ [24]. The LFL peptide was manufactured as described previously [24].

### 4.2. Samples

Samples of healthy volunteers’ peripheral blood were collected in vacuum tubes coated with EDTA. In total, 91 donors of a median age of 28, with a male/female rate of 41/50, participated in the study. The HCMV-specific IgG levels were measured in the plasma samples of healthy volunteers via an ELISA kit (Vector-Best, Novosibirsk, Russia). Prior to blood donation, donors gave informed consent for participation in the study, which was conducted according to guidelines of the Declaration of Helsinki and approved by the Ethics Committee of Pirogov Russian National Research Medical University (protocol #252 from 25 June 2025).

### 4.3. NK Cell Isolation

Peripheral blood mononuclear cells (PBMCs) were previously obtained from peripheral blood samples centrifuged in the Ficoll (PanEco) gradient, with a 1.077 g/cm^3^ density. Peripheral blood NK cells were isolated from PBMCs by a negative magnetic NK cell isolation kit (Miltenyi Biotech, Bergisch Gladbach, Germany) with a purity of >97%. NK cells after separation were ex vivo phenotyped or placed in culture plates in bulk cultures, sorted into subsets, and cultivated.

### 4.4. Bulk NK Cell Stimulation and In Vitro Expansion

Bulk NK cells were cultured in 96-well flat-bottom plates in the NK cell medium, which consisted of 45% NK MACS medium (Miltenyi Biotech), 10% FBS (HyClone), and DMEM medium, supplemented with 2mM L-glutamine–alanine (PanEco), 1% sodium pyruvate (PanEco), and 1% antibiotic–antimycotic (Millipore Sigma) at 37 °C in 5% CO_2_. IL-2 (Hoffmann La-Roche, Basel, Switzerland) was added to the medium at a concentration of 100 U/mL. Irradiated feeder cells, K562-mbIL21 or K562-21E, were preincubated with or without the LFL peptide and were added to NK cells at a ratio of NK:feeder = 1:1. An optimal concentration of 4–9 × 10^5^ cells/mL was maintained. Cell count was conducted on days 7 and 14 of cultivation using a cell counter (TC20, Bio-Rad Laboratories, Hercules, CA, USA); Trypan blue was added to the sample to exclude dead cells from analysis. The number of cells in 100-cell cultures was counted using a flow cytometer (MACSQuant 10 cytometer (Miltenyi Biotech) and LongCyte cytometer (ChallenBio, Beijing, China)); the cells were stained with SytoxBlue viability dye to exclude dead cells from analysis. The expansion coefficient was calculated as: K = N(t)/N(0), where N(t) is the cell count at time point t.

### 4.5. NK Cell Subset Stimulation and In Vitro Expansion

NK cell subset cultures were obtained immediately after negative magnetic separation using an FACSVantage/DiVa cell sorter (BD Biosciences, San Jose, CA, USA), equipped with lasers of 405, 488, and 643 nm. The instrument was calibrated prior to each sorting with QC beads; drop delay adjustment was performed using BD FACS Accudrop beads; and an additional calibration step included test plate sorting to ensure the correct cell distributions into plate wells. The sorting mode “Purity” allowed for obtaining cultures of high sorting purity at more than 95%. Prior to cell sorting, NK cells were labeled by Abs CD56-Brilliant Violet 421 (clone 5.1H11, Sony, San Jose, CA, USA), CD57-PE-Vio 770 (clone TB03, Miltenyi Biotech), KIR2DL2/3-FITC (clone REA1006, Miltenyi Biotech), and NKG2C-PE (clone REA205, Miltenyi Biotech) in a separation buffer (PanEco). CD57^−^KIR^−^NKG2C^−^, CD57^−^KIR^−^NKG2C^+^, CD57^−^KIR^+^NKG2C^−^, CD57^+^KIR^+^NKG2C^−^, and CD57^+^KIR^+^NKG2C^+^ NK cells were sorted into U-bottom 96-well plates, 100 cells per well (Figure S3). Three or five replicate cultures were performed. Culture medium of 100-cell cultures consisted of 20% X-vivo medium (Lonza, Walkersville, MD, USA) and 80% DMEM (PanEco), supplemented with 10% FBS, 2mM L-glutamine–alanine (PanEco), 1% sodium pyruvate (PanEco), and 1% antibiotic–antimycotic (Millipore Sigma). IL-2 was added at a concentration of 100 U/mL; feeder cells K562-mbIL21 and K562-21E, preincubated with or without LFL peptide, were added at a concentration of 10^4^ per mL. NK cell subset cultures were incubated for 3 weeks and counted on days 12 and 17.

### 4.6. Obtaining 3D Tumor Cultures

To form 3D cultures in vitro, 9-well gelled agarose plates were prepared as described previously [52]. The suspension of SKOV3 or MCF7 cells in DMEM medium supplemented with 10% FBS was added to the agarose wells (20,000 cells per well). The seeded pre-spheroids were incubated for 4–6 days in a CO_2_ incubator at 37 °C and in 5% CO_2_. The bulk NK cells or subset NK cells were added to spheroids at an E:T ratio of 1:1, and then cancer cells were incubated with NK cells for 24 h.

### 4.7. Spheroid Culture Viability

NK cell cytotoxicity against spheroid cultures was assessed by flow cytometry. Prior to analysis, spheroids with NK cells were washed with PBS (PanEco) and vigorously disintegrated by pipetting, then stained externally with the following anti-human antibodies (Abs): EpCAM-FITC (clone 028, SinoBiological, Beijing, China) and CD56-APC (clone REA196, Miltenyi Biotech). The cells of spheroids were distinguished as EpCAM^+^CD56^−^ via gating. The level of intracellular GrB was assessed using a kit for cellular fixation and intracellular staining (Inside Stain Kit, Miltenyi Biotech) with granzyme B AF647 Abs (clone GB11, Biolegend, San Jose, CA, USA). To assess the proportion of non-viable cells in spheroids, SytoxBlue viable dye (Invitrogen, Carlsbad, CA, USA) was added to samples immediately before the flow acquisition.

### 4.8. Cell Staining and Flow Cytometry

Flow cytometry data acquisition was performed using a MACSQuant 10 cytometer (Miltenyi Biotech) and LongCyte cytometer (ChallenBio, China), both equipped with 3 lasers of 408, 488, and 635 nm. Mouse anti-human fluorescent-labeled antibodies that were used for PBMC/NK cell surface staining were as follows: CD3-APC-Vio 770 (Clone REA613), CD56-FITC, CD56-PE-Vio 615, CD56-PE-Vio 770 (Clone REA196), CD57-VioBlue, CD57-APC-Vio 770 (Clone TB03), KIR2DL2/3-FITC, KIR2DL2/3-PE-Vio 615 (Clone REA 1006), KIR2DL2/3-APC (Clone DX27), NKG2A-PE, NKG2A-PE-Vio 770 (Clone REA110), NKG2C-FITC, NKG2C-PE (Clone REA205), HLA-DR-PE-Vio 770 (Clone REA805) (Miltenyi Biotech); CD3-PerCP (Clone HIT3a), CD56-Brilliant Violet 421 (Clone 5.1H11) (Sony, San Jose, CA, USA) (also shown in Table S1). The phenotyping included morphological gating in the FSC/SSC coordinates, followed by single-cell gating in the FSC-A/FSC-H coordinates. The ex vivo NK cell phenotype was observed in the PBMC fraction, where NK cells were determined as CD56^+^CD3^−^ cells and then phenotyped. The expanded NK cell phenotype was measured in the CD56-positive gate. No less than 50,000 events for NK cells in an FSC/SSC gate and no less than 10,000 events for NK cell subsets were recorded. Each experiment was performed using a compensation setting adjusted for a certain staining panel.

### 4.9. Degranulation Assay

To estimate the natural cytotoxicity of NK cell cultures, a degranulation assay was performed. NK cells were incubated in the NK cell medium without IL-2 for 24 h. Then, cells were stimulated with IL-2500 U/mL overnight. After stimulation, K562 cells were added to the NK cells at a ratio of E:T = 1:1 for 2.5 h in the presence of 2 μM monensin (Sigma-Aldrich, Burlington, MA, USA) and anti-CD107a antibody (CD107a-APC, Clone H4A3 (Sony)). The degranulation intensity was measured by the proportion of CD107a^+^ NK cells. For baseline degranulation controls, NK cells were incubated in the same way without K562 target cells.

### 4.10. Cytokine-Dependent IFNγ Production Assay

IFNγ production by NK cells was measured in subset cultures on day 17 of cultivation. NK cells were incubated in the culture medium without cytokines for 24 h. Then, NK cells were stimulated with 20 ng/mL of IL-12 and 20 ng/mL of IL-18 for 20 h, and 2 μM monensin was added 4 h before measurement. NK cells were stained with the CD56 antibody, fixed using an intracellular staining kit (Miltenyi Biotech), and stained with the IFNγ-PE antibody (clone 45-15, Miltenyi Biotech) (Table S1).

### 4.11. Statistical Analysis

Data were analyzed using GraphPadPrism (ver. 8.00, GraphPadSoftware, San Diego, CA, USA) and FlowJo VX (v10, FlowJo LLC, Ashland, OR, USA). Data distribution was assessed for normality using D’Agostino and Pearson’s test when sample size exceeded n = 8; for samples of 6 < n < 8, the Shapiro–Wilk test was used. Samples of n < 6 were considered non-normally distributed. The statistical significance of the obtained results was determined using unpaired and paired two-sample Student’s *t*-tests for normally distributed data; in other cases, the unpaired Mann–Whitney U test or paired Wilcoxon’s test was applied. Differences among the multiple groups were analyzed using the Kruskal–Wallis test for unpaired data or Friedman’s test for paired data, followed by Dunn’s multiple comparisons post hoc test to determine significant differences between specific pairs. Correlation analysis was performed using Pearson’s correlation for parametric samples and Spearman’s correlation for nonparametric samples. *p*-values of *p* < 0.05 were assumed statistically significant: * *p* < 0.05, ** *p* < 0.01, *** *p* < 0.001, **** *p* < 0.0001. The results are presented as means ± standard deviation unless otherwise noted.

## Data Availability

The data supporting the conclusions of this article will be made available by the authors upon request.

## Supplementary Materials

The following supporting information can be downloaded at: https://www.mdpi.com/article/10.3390/ijms27136087/s1.

## Abbreviations

The following abbreviations are used in this manuscript:

HCMV	Human Cytomegalovirus
KIR	Killer-cell immunoglobulin-like receptor
LFL	VMAPRTLFL peptide
