# Supplementary material for: HLA-E-Directed Accumulation of KIR+NKG2C+ NK Cells upon HCMV Peptide Presentation In Vitro: Association with the Ex Vivo Phenotype
Source: Int J Mol Sci. 2026 Jul 7;27(13):6087. doi: 10.3390/ijms27136087 (PMC13362507; doi:10.3390/ijms27136087)
Supplement: Supplementary file 1 [file ijms-27-06087-s001.zip › Supplementary figures and table.pdf]

Supplementary materials

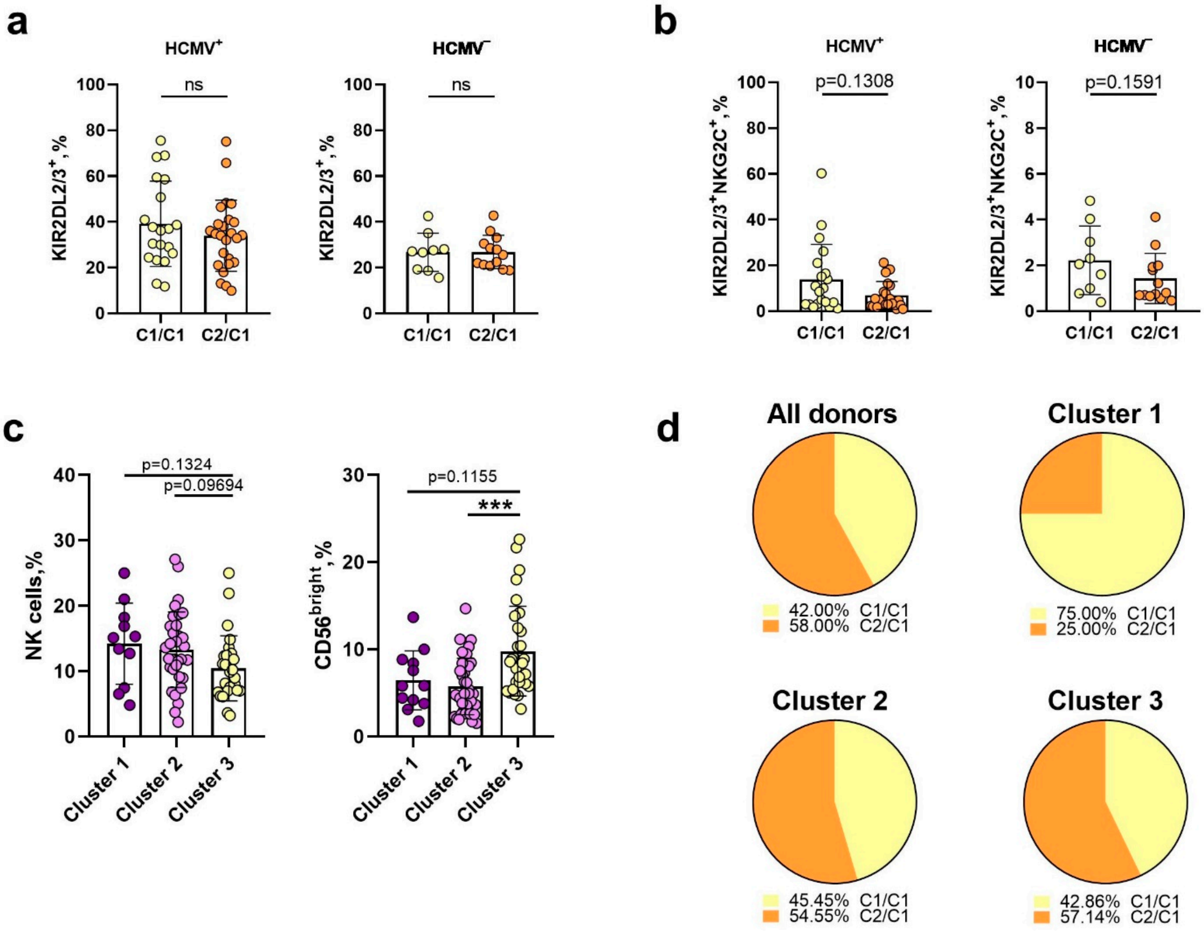

Figure S1. The connection between phenotype of NK cells and donor's HLA-C genotype. (**a**) The proportion of KIR$^+$ cells in total NK cell population of HCMV$^+$ (left) and HCMV$^-$ (right) individuals with one (C2/C1) and two (C1/C1) HLA-C1 alleles; (**b**) The proportion of KIR+NKG2C+ cells in total NK cell population of HCMV$^+$ (left) and HCMV$^-$ (right) individuals with one (C2/C1) and two (C1/C1) HLA-C1 alleles; (**c**) The proportion of NK cells and CD56bright cells in the NK cell population of different clusters' donors; (**d**) The proportion of donors bearing one (C2/C1) or two (C1/C1) HLA-C1 allele in total population excluding donors with HLA-C2/C2 genotype and in three clusters. Statistical analysis was performed using nonparametric non-paired Mann-Whitney U-test (*** p<0.005), means±SD are shown.

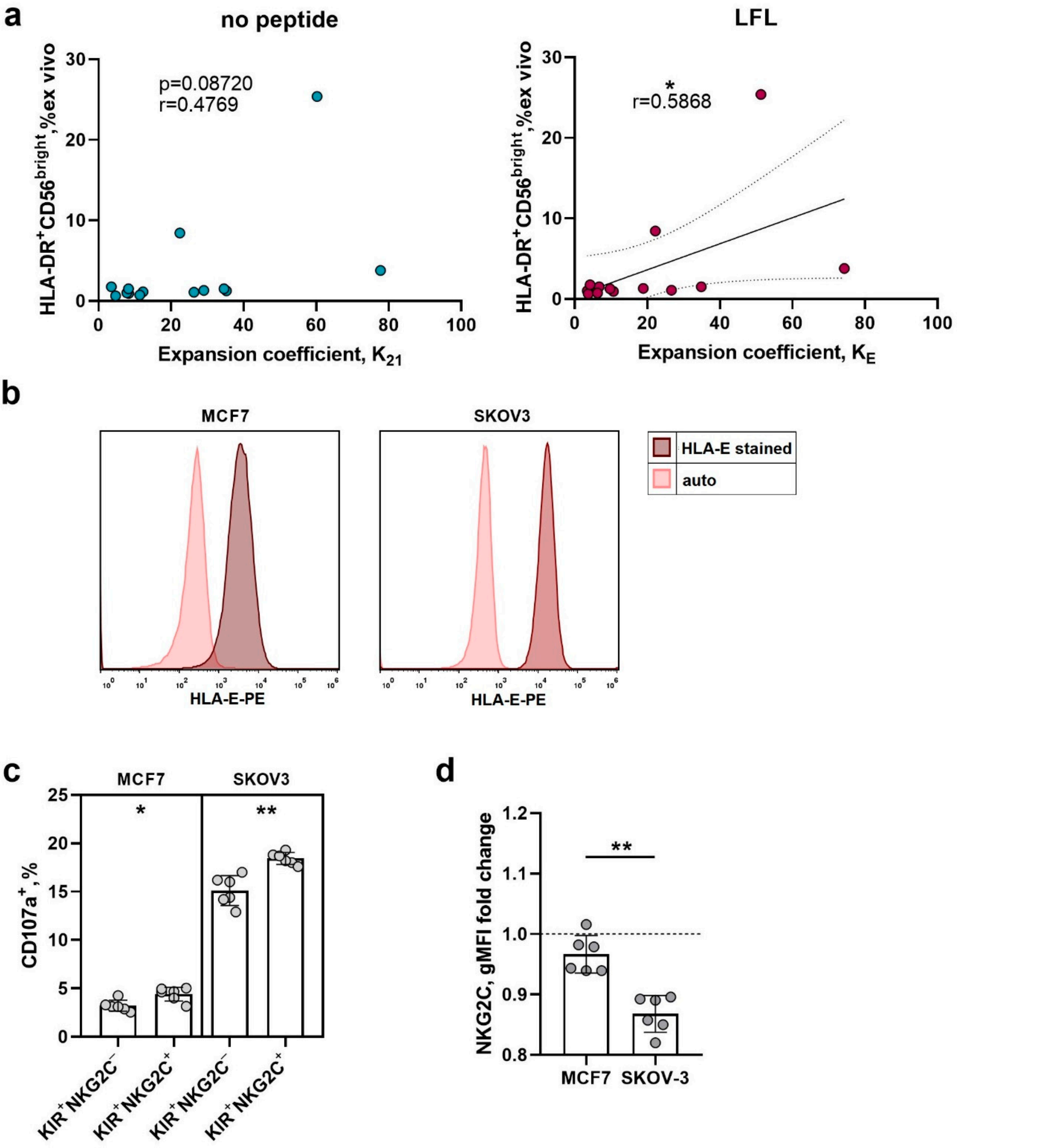

Figure S2. Proliferative activity of bulk NK cell cultures obtained with and without LFL peptide presentation and HLA-E-targeted cytotoxicity of NK cells. (**a**) Correlation between the expansion coefficient of NK cell cultures obtained without (left) and with (right) LFL peptide presentation and the proportion of HLA-DR^{+}CD56^{bright} NK cells ex vivo; (**b**) The expression of HLA-E by the MCF7 (gMFI = 34.3) and SKOV-3 (gMFI = 11.9) cells lines; (**c**) The proportion of CD107a^{+} degranulating cells KIR^{+} NK cells with different expression of NKG2C after incubation with MCF7 and SKOV-3 spheroids; (**d**) The expression intensity of NKG2C in bulk NK cell cultures after incubation with MCF7 and SKOV-3 spheroids. Statistical analysis was performed using a nonparametric Mann-Whitney test (* $p < 0.05$, ** $p<0.01$), means±SD are shown. Correlation analysis was done using Pearson correlation, $p<0.05$ was considered statistically significant.

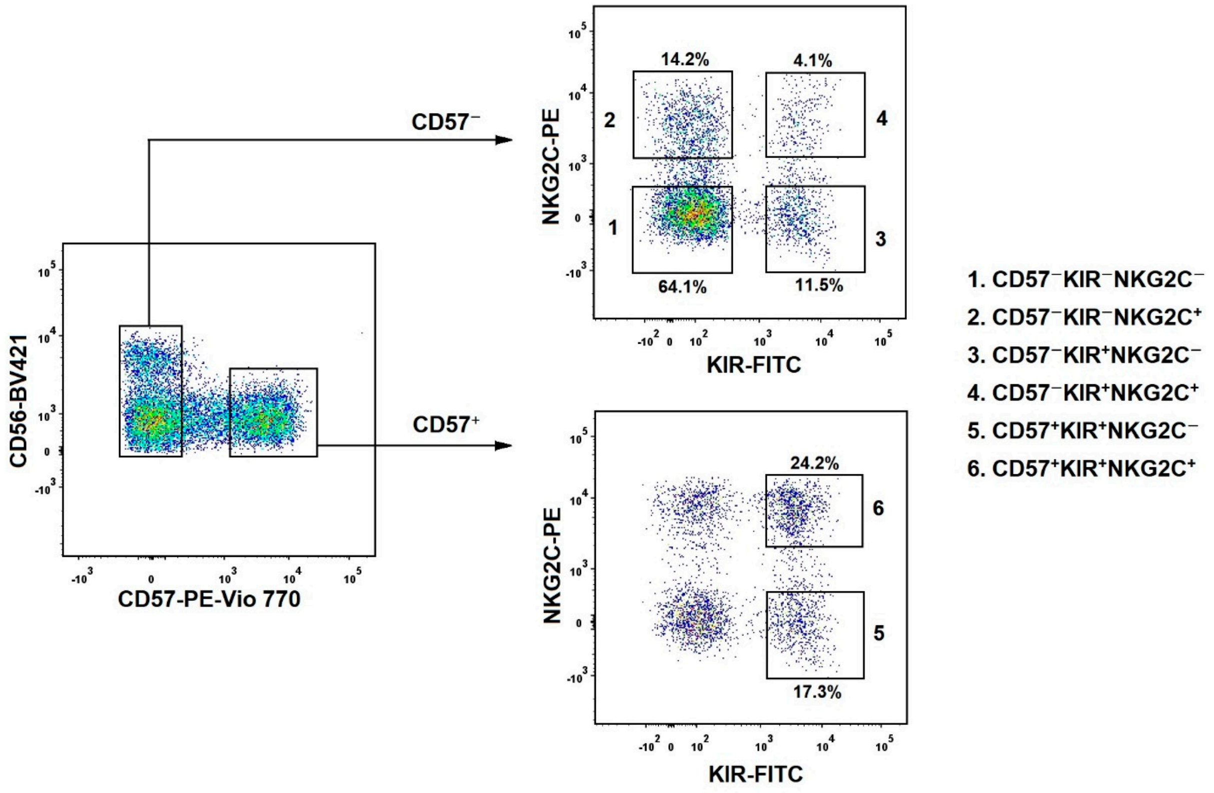

Figure S3. Gating scheme of cell sorting for collection of NK cell subsets.

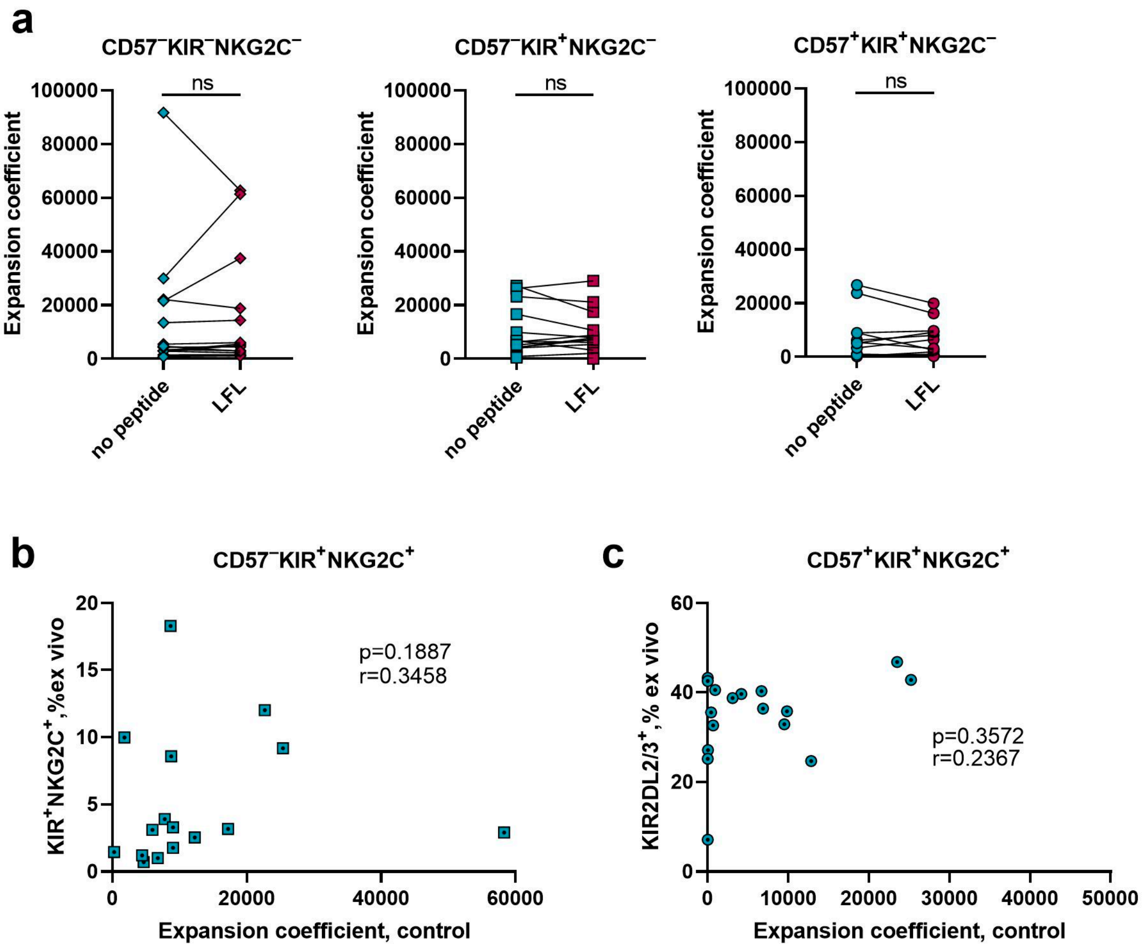

Figure S4. Proliferative activity of NK cell subsets with different expression of KIR, NKG2C, and CD57 cultured with or without LFL presentation. (**a**) Expansion coefficient of $NKG2C^{-}$ cultures with different KIR and CD57 expression, which were cultured with or without LFL peptide presentation; (**b**) Correlation between the expansion coefficient of $CD57^{-}KIR^{+}NKG2C^{+}$ cells control cultures and the proportion of $KIR^{+}NKG2C^{+}$ NK cells ex vivo; (**c**) Correlation between the expansion coefficient of $CD57^{+}KIR^{+}NKG2C^{+}$ cells control cultures and the proportion of NK cells KIR+ ex vivo. Duration of cultivation T = 12 days, number of donors N = 17 ($HCMV^{+}$ - 11, $HCMV^{-}$ - 6). Statistical analysis was performed using a nonparametric Wilcoxon test (a). Correlation analysis was done using Pearson correlation, $p<0.05$ was considered statistically significant.

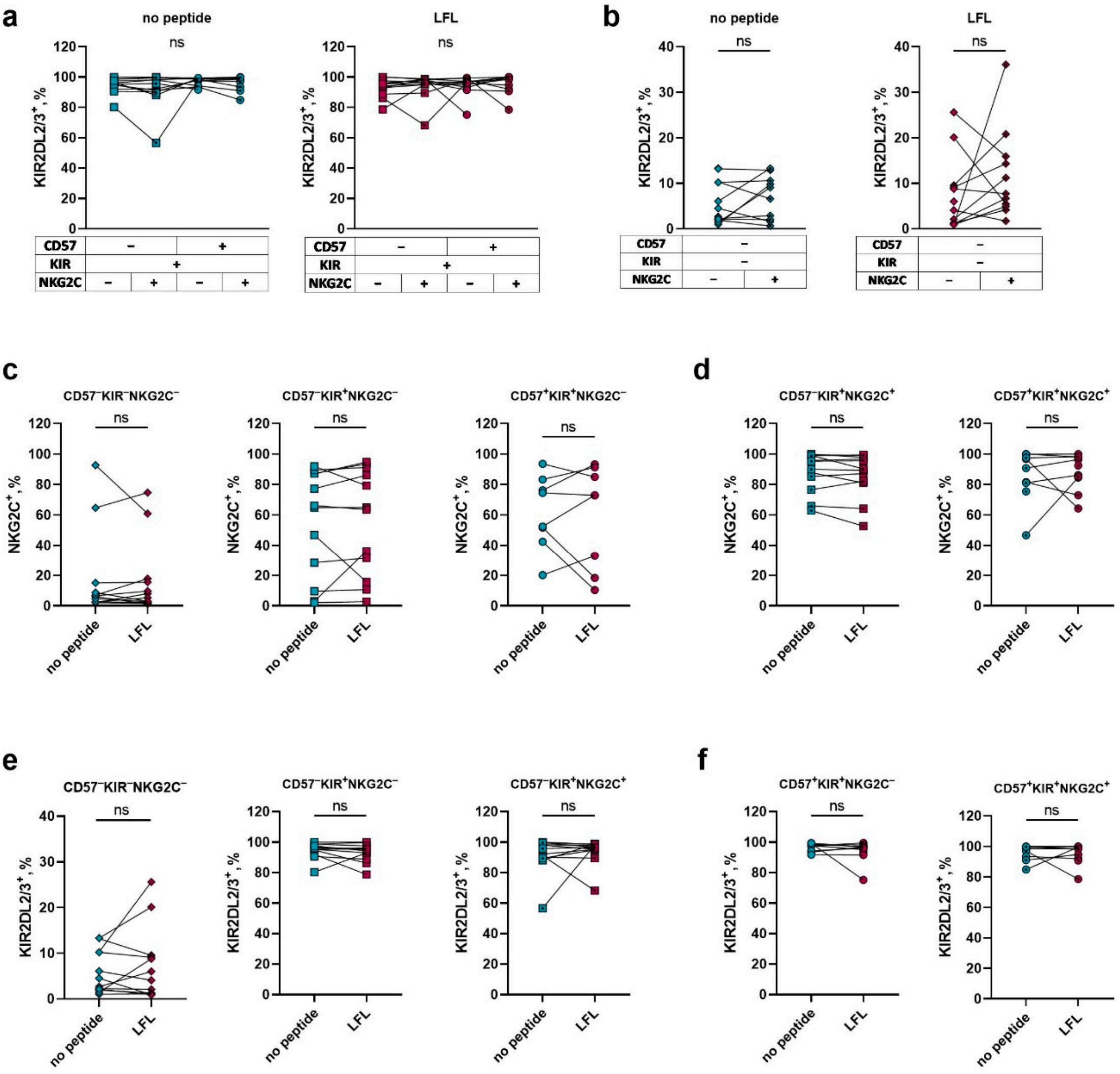

Figure S5. Expression stability and de novo expression of KIR and NKG2C in NK cell subset cultures with different expression of KIR, NKG2C and CD57, activated in the presence or absence of LFL peptide. (**a**) Stability of KIR expression in cultures of KIR-positive subsets with different expression of NKG2C and CD57, activated in the presence (right) or absence (left) of LFL peptide; (**b**) De novo KIR expression in cultures of $CD57^-KIR^-$ subsets with or without NKG2C expression, activated in the presence (right) or absence (left) of LFL peptide; (**c**) De novo NKG2C expression in cultures of NKG2C-negative subsets with different expression of KIR and CD57, activated in the presence or absence of LFL peptide; (**d**) NKG2C expression stability in cultures of KIR^+NKG2C^+ subsets with different expression of CD57, activated in the presence or absence of LFL peptide; (**e**) De novo KIR expression and KIR expression stability in cultures of CD57-negative subsets with different expression of KIR and NKG2C, activated in the presence or absence of LFL peptide; (**f**) KIR expression stability in cultures of $CD57^+KIR^+$ subsets with different expression of NKG2C, activated in the presence or absence of LFL peptide. Statistical analysis was performed using a nonparametric Wilcoxon test.

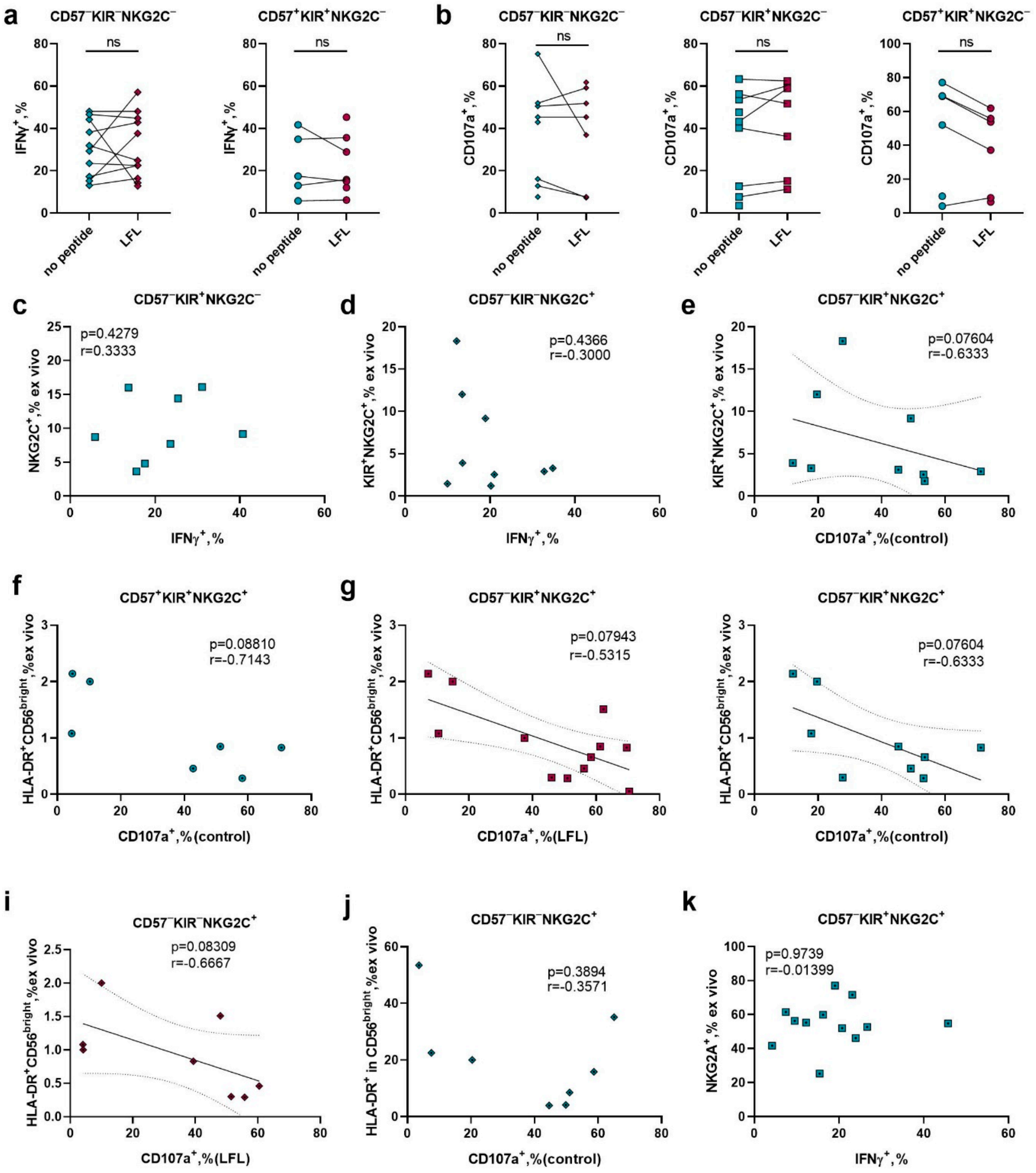

Figure S6. Functional activity of NK cell subset cultures with different expression of KIR, NKG2C, and CD57, activated in the presence or absence of LFL peptide. (**a**) Proportion of IFNγ-producing NK cells in cultures of NKG2C-negative subsets with different expression of KIR and CD57; (**b**) Proportion of degranulating CD107a^{+} NK cells in cultures of NKG2C-negative subsets with different expression of KIR and CD57 upon coincubation with K562 targets; (**c**) Correlation between the proportion of IFNγ-producing cells in CD57^{-}KIR^{+}NKG2C^{-} subset control cultures and the proportion of NK cells NKG2C^{+} ex vivo; (**d**) Correlation between the proportion of IFNγ-producing cells in CD57^{-}KIR^{-}NKG2C^{+} subset control cultures and the proportion of NK cells KIR^{+}NKG2C^{+} ex vivo; (**e**) Correlation between the proportion of IFNγ-producing cells in CD57^{-}KIR^{+}NKG2C^{+} subset control cultures and the proportion of NK cells KIR^{+}NKG2C^{+} ex vivo; (**f**) Correlation between the relative proportion of CD107a^{+} cells in CD57^{+}KIR^{+}NKG2C^{+} subset

control cultures and the proportion of HLA-DR$^+$CD56^{bright} NK cells ex vivo; (**g**) Correlation between the relative proportion of CD107a$^+$ cells in CD57-KIR+NKG2C+ subset cultures presented with LFL peptide and the proportion of HLA-DR$^+$CD56^{bright} NK cells ex vivo; (**h**) Correlation between the relative proportion of CD107a$^+$ cells in CD57$^-$KIR$^+$NKG2C$^+$ subset control cultures and the proportion of HLA-DR$^+$CD56^{bright} NK cells ex vivo; (**i**) Correlation between the relative proportion of CD107a$^+$ cells in CD57$^-$KIR$^-$NKG2C$^+$ subset cultures presented with LFL peptide and the proportion of HLA-DR$^+$CD56^{bright} NK cells ex vivo; (**j**) Correlation between the relative proportion of CD107a$^+$ cells in CD57$^-$KIR$^-$NKG2C$^+$ subset control cultures and the proportion of HLA-DR$^+$CD56^{bright} NK cells ex vivo; (**k**) Correlation between the proportion of IFNγ-producing cells in CD57$^-$KIR$^+$NKG2C$^+$ subset control cultures and the proportion of NKG2A$^+$ NK cells ex vivo. Duration of cultivation T = 17 days, number of donors N = 17 (HCMV$^+$ - 11, HCMV$^-$ - 6). Statistical analysis was performed using a nonparametric Wilcoxon test (* $p< 0.05$). Correlation analysis was done using Pearson correlation, $p<0.05$ was considered statistically significant.

Table S1. Anti-human antibody list.

Antibody	*Fluorophore*	*Clone*	*Source*	*Source location*
CD3	APC-Vio 770	REA613	Miltenyi Biotech	Bergisch Gladbach, Germany
CD3	PerCP	HIT3a	Sony	San Jose, CA, USA
CD56	Brilliant Violet 421	5.1H11	Sony	San Jose, CA, USA
CD56	FITC	REA196	Miltenyi Biotech	Bergisch Gladbach, Germany
CD56	PE-Vio 615	REA196	Miltenyi Biotech	Bergisch Gladbach, Germany
CD56	PE-Vio 770	REA196	Miltenyi Biotech	Bergisch Gladbach, Germany
CD57	VioBlue	TB03	Miltenyi Biotech	Bergisch Gladbach, Germany
CD57	APC-Vio 770	TB03	Miltenyi Biotech	Bergisch Gladbach, Germany
CD107a	APC	H4A3	Sony	San Jose, CA, USA
EpCAM	FITC	028	SinoBiological	Beijing, China
GrB	AF647	GB11	Biolegend	San Jose, CA, USA
IFNγ	PE	45-15	Miltenyi Biotech	Bergisch Gladbach, Germany
KIR2DL2/3	FITC	REA1006	Miltenyi Biotech	Bergisch Gladbach, Germany
KIR2DL2/3	PE-Vio615	REA1006	Miltenyi Biotech	Bergisch Gladbach, Germany
KIR2DL2/3	APC	DX27	Miltenyi Biotech	Bergisch Gladbach, Germany
NKG2A	PE	REA110	Miltenyi Biotech	Bergisch Gladbach, Germany
NKG2A	PE-Vio 770	REA110	Miltenyi Biotech	Bergisch Gladbach, Germany
NKG2C	FITC	REA205	Miltenyi Biotech	Bergisch Gladbach, Germany
NKG2C	PE	REA205	Miltenyi Biotech	Bergisch Gladbach, Germany
HLA-DR	PE-Vio 770	REA805	Miltenyi Biotech	Bergisch Gladbach, Germany
